# A co-created nurse-driven catheterisation protocol can reduce bladder distension in acute hip fracture patients - results from a longitudinal observational study

**DOI:** 10.1186/s12912-022-01057-z

**Published:** 2022-10-12

**Authors:** Maria Frödin, Bengt Nellgård, Cecilia Rogmark, Brigid M. Gillespie, Ewa Wikström, Annette Erichsen Andersson

**Affiliations:** 1 Institute of Health and Care Sciences, Sahlgrenska Academy, University of Gothenburg, Gothenburg, Sweden; 2 Department of Anaesthesiology and Intensive Care Medicine, Institute of Clinical Sciences, Sahlgrenska University Hospital, Ortopedoperation 1, Göteborgsvägen 31, SE-431 80 Gothenburg, Sweden; 3 Skane University Hospital, Department of Orthopaedics Malmö, Lund University, Lund, Sweden; 4 The Swedish Arthroplasty Register, Registercentrum VGR, Gothenburg, Sweden; 5 NMHRC Centre of Research Excellence in Wiser Wound Care, Menzies Health Institute, Queensland, Griffith University, Brisbane, Australia; 6 Gold Coast University Hospital and Health Service, Southport, Australia; 7 School of Business, Economics and Law, Department of Business Administration, University of Gothenburg, Gothenburg, Sweden

**Keywords:** Nurse-driven urinary catheterisation protocol, Bladder distension, Urinary retention, Hip fracture

## Abstract

**Background:**

Urinary retention is common in elderly patients undergoing acute hip fracture surgery. Avoiding overfilling the urinary bladder is important to avoid detrusor muscle damage and associated motility problems. The aim of this study was to analyse associations between the co-creation of a nurse-driven urinary catheterisation protocol and the incidence of bladder distension in patients undergoing hip fracture surgery.

**Methods:**

This is a single-centre implementation intervention with a retrospective longitudinal observation design, using five measures points, spanning from June 2015 to March 2020. The intervention was theory driven and the participants, together with the facilitators and researcher, co-created a nurse-driven urinary catheterisation protocol. Data were retrieved from the hip fracture register. Uni- and multivariable logistic regressions were used for analyses of changes in bladder distension and urinary volume of ≥500 ml over the years.

**Results:**

A total of 3078 patients were included over a five-year period. The implementation intervention was associated with a reduction in the proportion of patients with bladder distension of 31.5% (95% confidence interval 26.0–37.0), from year 1 to year 5. The multivariable analysis indicated a 39% yearly reduction in bladder distension, OR 0.61 (95% confidence interval 0.57–0.64, *p* <  0001). There was a reduction in the proportion of patients with a bladder volume of ≥500 ml of 42.8% (95% confidence interval 36.2–49.4), from year 1 to year 5. The multivariable analysis found a 41% yearly reduction in patients with a bladder volume of ≥500 ml, OR 0.59 (95% confidence interval 0.55–0.64, *p* <  0.0001). The intervention was associated with improved documentation of both catheter indications and removal plans.

**Conclusion:**

The use of predefined catheter indications and a tighter bladder scanning schedule were associated with a reduction in the incidence of both bladder distension and urine volume ≥ 500 ml in hip fracture patients. Registered nurses can play an active role in the facilitation of timely and appropriate catheter treatment in patients with hip fractures.

**Trial registration:**

Clinical Trial Registry ISRCTN 17022695 registered retrospectively on 23 December 2021, in the end of the study, after data collection.

**Supplementary Information:**

The online version contains supplementary material available at 10.1186/s12912-022-01057-z.

## Background

Since To Err is Human report [1], healthcare has a good knowledge of hospital-related adverse events, their associated preventive strategies, but also how challenging it can be to implement and routinise evidence-based best practice in clinical daily work [2, 3]. In high-income countries, one in ten patients is estimated to suffer from adverse events [3]. In Sweden, adverse events occurs in almost 98,000 patients every year, of which bladder distension was reported in approximately 10% of the adverse events [4]. Further, bladder distension is a largely preventable adverse event if evidence-based best practice is adhered to [4, 5]. Bladder distension occurs when the bladder is overfilled with urine. Even though the bladder threshold varies [6], and reduces with age [7], bladder capacity is commonly reported to range between 400 and 600 ml and, in some patients, a volume of between 500 and 1000 ml might be unharmful, if treated within one to 2 hours [8, 9], However, if undetected, the tension of the bladder wall when it is overfilled can damage the detrusor muscle [8, 10, 11]. Iatrogenic bladder damage has been shown to affect patients’ daily life substantially, due to chronic catheter treatment or straight in-out self-catheterisation, recurrent urinary tract infections (UTIs) and/or urosepsis and a mistrust to the healthcare system [12]. Moreover, insufficient routines, lack of knowledge and poor communication between healthcare workers (HCW) and patients have been identified as factors contributing to the development of bladder damage [12].

Orthopaedic patients are especially prone to develop bladder distension compared to other specialities [5, 13]. Specifically, most patients undergoing hip fracture surgery, have several intrinsic and extrinsic factors that increase the risk of urinary retention (UR) [7, 8, 14, 15]. The reported incidence of pre- and post-operative UR varies between 4 and 82% [16–23] and has proven to be a persistent problem in rehabilitation units [24]. Variations in UR rates may explain some of these findings.

In 2015, we initiated the Safe Hands project [25, 26], where new preventive bundle routines were co-created with HCW. One routine aiming to improve hand hygiene, the use of aseptic insertion techniques and indwelling urinary catheter (IDC) care was associated with a reduction in urinary catheter (UC)-associated UTI from 18 to 4%, after introduction in the care pathway of hip fracture patients, which was the *first step* in our bladder bundle [27]. As a co-finding, we observed a high incidence of bladder distension, a lack of appropriate use of IDC indications removal plan and related documentation, as well as a timely bladder scan. As a result, increased awareness and the use of preventive strategies were needed [28–33]. Given this, a joint decision was taken by managers, leaders, quality co-ordinators and researchers to also address these problems by including a co-created nurse-driven UC protocol and timely bladder scanning schedule as a *second step* in our bladder bundle intervention. The overall aim of this study was to analyse association between the co-creation of a nurse-driven UC protocol and the incidence of bladder distension in patients undergoing hip fracture surgery.

## Methods

### Design

This is a single-centre implementation intervention with a retrospective longitudinal observation design, using five measures points, spanning from June 2015 to March 2020. For patient outcomes, data from the local hip fracture quality register were retrieved and analysed. The primary outcome: Changes in the incidence of bladder distension before and after the intervention. Bladder distension were defined as; a) urine volume ≥ 500 ml twice or ≥ 1000 ml once, according to the Swedish national trigger tool [34], b) a physician-diagnosed bladder distension with a urine volume of < 1000 ml or no volume documented and with an IDC present at discharge. *Not defined as bladder distension*; patients with a documented urine volume of < 1000 ml once or no volume documented, treated with an IDC due to indications other than UR or residual urine.

Secondary outcomes: Changes in the incidence before and after the intervention of, i) a bladder volume of ≥500 ml, ii) the largest urine volume documented during hospital stay and iii) changes in documented catheter indication(s) and removal plan over the years. Patients with no urine volume documented and an IDC indication other than UR or residual urine were considered as not having a urine volume of ≥500 ml. The Strengthening the Reporting of Observational studies in Epidemiology (STROBE) criteria for reporting observational studies were followed [35].

### Setting and participants

The study setting was an orthopaedic department at a university hospital performing 800–900 acute hip fracture surgeries annually. The participating wards were selected as they were involved in the care of acute hip fracture patients, ≥ 65 years of age: the emergency department (ED), three ortho-geriatric wards, the operating room (OR) and the post-anaesthesia care unit (PACU)/intensive care units (ICU). The HCW participating in the intervention program were registered nurses (RNs) approximately 400, of which some were specialist within critical care, anestesia care nurses, OR-nurses and within surgical care and nurse assistants. Appointed local facilitators, called expert nurses, participated in the learning lab meetings describe below. The register nurses in the involved units assessed patients with hip fracture according to the protocol, described below. These patients did not participate in the intervention program.

Prior to the intervention, the hospital’s routine for preventing UTI was to use straight in-out catheterisation if the UR was ≥400 ml, with a six- to eight-hour bladder scanning timespan to measure UR or residual urine. An IDC was used, if prescribed by a physician, and routinely removed on day one post-surgery, unless a need to continue was identified, using an IDC marker on the patient board alerting *that* an IDC was in situ.

### Theoretical foundation and implementation strategies

This study was based on integrated knowledge translation (iKT) to facilitate knowledge transfer, i.e. the researcher and main facilitators work in partnership and collaborate with the local expert nurses during the implementation period [36]. The iKT processes used were informed by theories of dialogue and organisational learning [37, 38]. Facilitation was used as a means of overcoming barriers and supporting the participants [39, 40]. The implementation process had an emergent and flexible approach. The main facilitators were:

i. a senior researcher, RN and expert in infection prevention and implementation
ii. an RN specialising in critical care and anaesthesia nursing
iii. a senior researcher, consultant specialist in anaesthesiology and expert on hip fracture patients
iv. a consultant specialist in gerontology

**Fig. 1:**
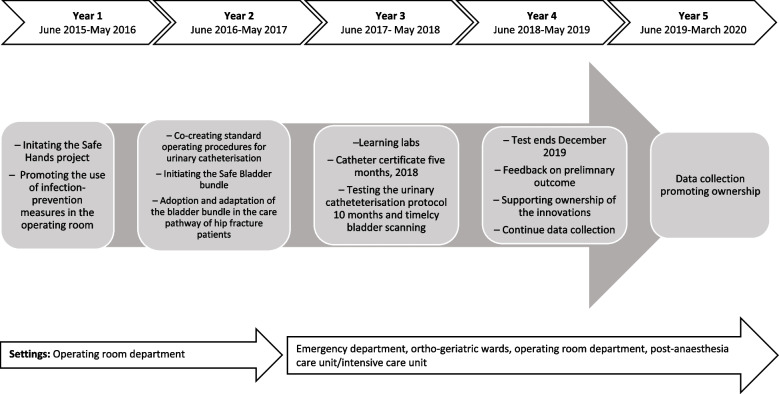
Time frame and intervention components

As part of the iKT process, the main facilitators and research together with the local expert nurses, appointed physicians, first-line leaders and quality co-ordinators set out goals and plans for the intervention. The local expert nurses were appointed by the first-line leaders, either one to two RNs and/or nurse assistants, who functioned as internal facilitators. Moreover, in the pre-planning of the intervention, potential barriers and enablers were considered, as well as specific contextual features [41, 42]. The time frame of the implementation intervention, strategies and components are presented in Fig. 1.

### Interventional components and activities

The facilitators held several educational meetings at the involved units and with the expert nurses. The meetings with the expert nurses took place in *learning labs,* which aimed to create a safe place for learning together through dialogue [37, 38]. See Table 1 for the educational components and implementation process, Additional file 1 for a patient case used for educational purposes and Additional file 2 for a brief version of the protocol.

**Table 1:** Description of the content in the learning labs and the development of the urinary catheterisation protocol

**Learning lab content**	
– Lecture on infection prevention, fluid balance and pre-optimisation in elderly patients. – Expert nurses and researcher review the literature. –Refresh assessment and communication tools: SBAR [43–45], early warning score and triage tool^a^ [46–48], ABCDE + F [49, 50], specifically related to patients’ need for catheterisation or not, CRM: decision-making, situational awareness and prioritisation related to urinary catheterisation [51, 52]. – Encourage thinking together i.e. ask a peer or physician for support in the decision process if needed, as a sign of growth, not a weakness. – Dialogue relating to: i. Evidence-based catheter indications and removal plan and alternative to IDC such as suprapubic catheter. ii. Patient cases. iii. The risk of self-termination of invasive devices, which can occur in patients with cognitive dysfunction or acute confusion. iv. The use of straight in-out catheterisation or alternative to indwelling urinary catheter. v. Appropriate documentation to prevent the loss of information. vi. Patient involvement i.e. to see them as competent individuals and experts on their body and function [53].	
**Practical procedure**	
– Co-creating the nurse-driven urinary catheterisation protocol. – Introduce the national schedule for measuring residual urine via a portable bladder scanner, adapted to fit the study site bladder volume threshold of ≥400 ml, starting on admission [54], see below. – If in need of straight in-out catheterisation before transport to the pre-operative area, or a pre-operative urine volume of ≥200 ml before start of anaesthesia and anticipated > 3 hours to end of surgery, insert an indwelling catheter and remove within 24–48 hours. – If no catheter, perform bladder scan immediately at the end of surgery, after wound closure and continuous post-operatively according to the schedule. – Use a catheter with a thermistor to facilitate peri-operative measurement of patients’ temperature. – Document indication, removal plan and perform a daily evaluation for catheter placed > 48 hours. – Developed pocket-sized stickers with indication, removal plan and scanning schedule.	
**Bladder scanning schedule** Residual urine: 100–150 ml – control after three hours 150–300 ml – control after two hours 300–400 ml – control after one hour ≥400 ml – perform straight in-out catheterisation or indwelling catheter depending on patient assessment, patient involvement and the further care plan	

*Abbreviations*: *SBAR* Situation, Background, Assessment, Recommendation, *ABCDE + F* A = airway, B = breathing, C = circulation, D = disability, E = exposure, F = further care, *CRM* Crew resource management, *IDC* Indwelling urinary catheter

^a^Early warning score: (MEWS): Modified Early Warning Score and (NEWS 2): National Early Warning Score. RETTS (Rapid Emergency Triage and Treatment System)

### The implementation process of the nurse-driven urinary catheterisation protocol

For 10 months, RNs consecutively assessed patients with hip fractures, ≥ 65 years of age, on admission, according to the UC protocol. The protocol followed the patients until discharge. The RNs were encouraged to consult a colleague, the expert nurses, or a physician if in doubt about indication(s) or catheter removal plan. A weekly evaluation of adherence to the protocol was performed by two of the main facilitators. Direct feedback was given to the RN, expert nurse and participating physicians, if any incorrect assessments were identified.

### Data collection

Outcome data were extracted from the hospital-based quality register of orthogeriatric hip fractures. Patients were included in the register by the discharge nurse. Thereafter the data were validated against the electronic medical records by a research nurse, a senior nurse anaesthetist specialist in infection control. We used the same exclusion criteria from the first step of our bladder bundle [27]. Patients with a hospital stay of ≤2 days, distal fracture, resection arthroplasty or previously included due to contralateral hip fracture, no catheterisation/chronic catheter/suprapubic/urostomy/dialysis, or straight in-out catheterisation/self-catheterisation were excluded.

The extracted variables were age, gender, ASA-classification score I-IV [55], hospital length of stay (LOS), diabetes mellitus type I and II, type of catheterisation treatment (indwelling, straight in-out or both), catheter days (including re-catheterisation days), documented catheter indication and removal plan, number of straight in-out catheterisations, re-catheterisation catheter present at discharge, largest bladder volume documented during hospital stay, urine volume ≥ 500 ml (yes/no) and bladder distension (yes/no). The hospital procedure was to measure the volume after catheterisation either by reading the marker on the urine bag or by pouring it into a litre measuring cup.

### Assessment of the protocol nurse-driven urinary catheterisation protocol

The data from the nurse-driven protocol was descriptively described using numbers and percentages. We used both RN documentation in the UC protocol and the electronical medical records for assessing adherence to the protocol. If correct IDC indication(s) and/or removal plan were identified in the electronical medical records but not in the protocol, this was counted as a successful identification and vice versa. If they were correct but differed, they were assessed as more than one indication. If one or more were not an appropriate indication this was assessed as incorrect indication(s). If the RN had documented *remove after surgery* as removal plan, we assessed it as correct even though it was not pre-defined removal plan, and removal plan *uncertain* was assessed as incorrect. Timely insertions were assessed by setting for first IDC insertion and changes in the number of patients treated with both IDC and straight in-out catheterisation.

### Statistical analysis

The categorical variables were presented as numbers and percentages and continuous variables as means, standard deviation or median, quartile 1 and quartile 3. For ordered group comparisons, the Mantel-Haenszel chi-square test was used for ordered categorical variables and dichotomous variables and Jonckheere-Terpstra test for continuous variables. For comparisons between two groups, Fisher’s exact test was used for dichotomous variables and Fisher’s non-parametric permutation test was used for continuous variables. Mean changes between year 1 and year 5 with 95% CI are given for bladder distension, a urine volume ≥ 500 ml and the largest observed urine volume. The confidence interval for dichotomous variables was the unconditional exact confidence limits and, if no exact limits could be computed, the asymptotic Wald confidence limits with continuity correction were calculated instead. The confidence interval for the mean difference between groups was based on Fisher’s non-parametric permutation test. Univariable logistic regression analysis was performed. Multivariable logistic regression was used to analyse the effectiveness of the intervention over years on bladder distension and a urine volume ≥ 500 ml, with adjustment for ASA-classification score, age, gender, LOS and diabetes. The results are given as odds ratio (OR) with 95% confidence interval (CI). To describe the goodness of fit of the model, we calculated the area under the ROC curve (AUC) [56]. All significance tests were two-sided and conducted at the 5% significance level. SAS version 9.4 was used for all these analyses [57].

## Results

### Primary outcome

**Fig. 2:**
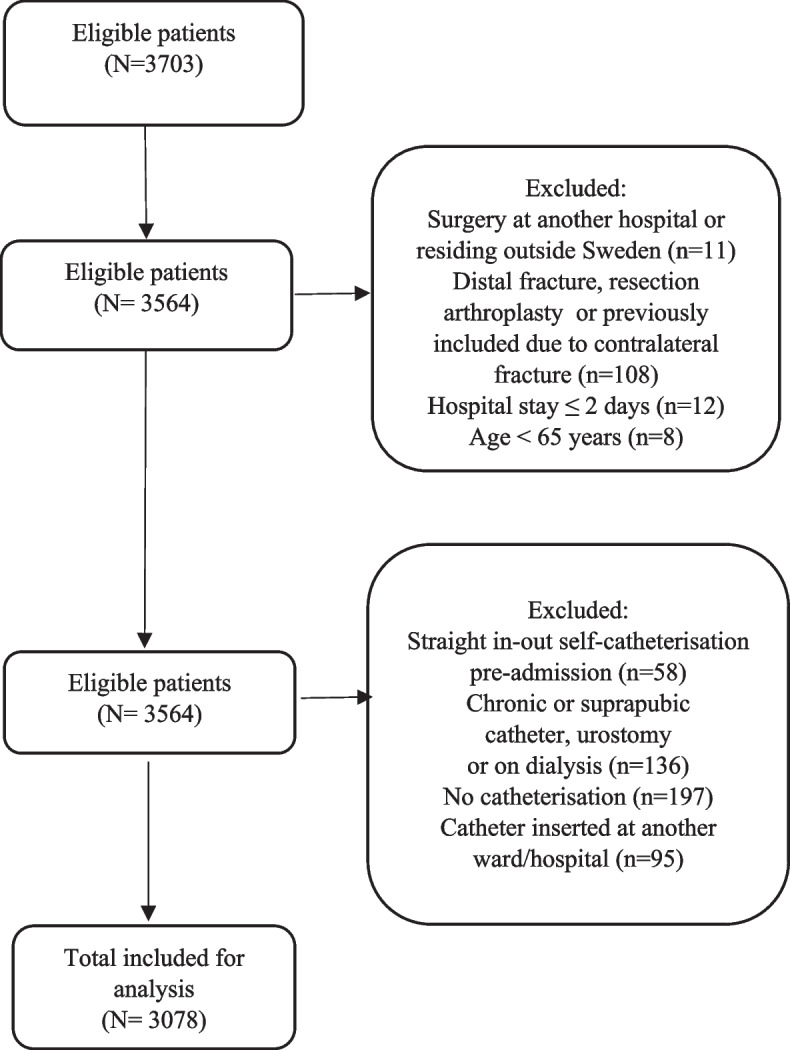
Flowchart of included and excluded patients from June 2015–March 2020

Data from 3078 patients were assessed for yearly incidence of bladder distension over 5 years, see Fig. 2 for the excluded 625 patients. Patient demographics did not differ over the years (Table 2). We observed a reduction in hospital LOS of 5 days from year one to year five (Table 2).

**Table 2:** Patient and clinical characteristic, *N* = 3078

Variables	Year 1, June 2015-May 2016	Year 2, June 2016-May 2017	Year 3, June 2017-May 2018	Year 4, June 2018-May 2019	Year 5, June 2019 March 2020	***p*** Over the periods
Patients’ characteristics	***n*** = 406	***n*** = 655	***n*** = 700	***n*** = 691	***n*** = 626	
Age, years	83.8 (8.0), 85 (79; 90)	84.8 (7.7), 86 (80; 91)	83.9 (8.3), 85 (78; 90)	83.9 (8.3), 85 (78; 90)	83.6 (8.2), 84 (77; 90)	0.091
Gender, female	294 (72.4)	446 (68.1)	493 (70.4)	501 (72.5)	443 (70.8)	0.63
ASA						
I	15 (3.7)	14 (2.1)	11 (1.6)	29 (4.2)	16 (2.6)	
II	165 (40.6)	264 (40.3)	257 (36.7)	286 (41.4)	258 (41.2)	
III	196 (48.3)	333 (50.8)	390 (55.7)	343 (49.6)	312 (49.8)	
IV	30 (7.4)	44 (6.7)	42 (6.0)	33 (4.8)	40 (6.4)	0.34
Diabetes mellitus	57 (14.0)	105 (16.0)	102 (14.6)	108 (15.6)	104 (16.6)	0.38
**Clinical characteristics**						
Hospital length of stay, days	14.7 (7.2) 14 (10; 17)	13.4 (7.7) 13 (8; 17)	12.2 (6.4) 11 (8; 15)	10.6 (5.2) 10 (7; 13)	9.75 (4.4) 9 (7; 12)	< 0.0001

For categorical variables, n (%) is presented

For continuous variables, the mean (SD)/median (Q1; Q3)/*n* = is presented

For comparisons between ordered groups, the Mantel-Haenszel chi-square test was used for ordered categorical and dichotomous variables. The Jonckheere-Terpstra test was used for continuous variables

Bladder distension was reduced over the years, from 40.6% in year 1 to 9.1% (*p* < 0.0001) in year 5 (Table 3). The mean difference over the years in bladder distension were 31.5 (95% CI 26.0; 37.0).

**Table 3:** Catheterisation characteristics and documentation from year 1 to year 5

Variables	Year 1, June 2015-May 2016	Year 2, June 2016-May 2017	Year 3, June 2017-May 2018	Year 4, June 2018-May 2019	Year 5, June 2019-March 2020	***p*** Over the periods
	***n*** **= 406**	***n*** **= 655**	***n*** **= 700**	***n*** **= 691**	***n*** **= 626**	
**Bladder distension**	165 (40.6)	211 (32.2)	154 (22.0)	84 (12.2)	57 (9.1)	<.00001
Urine volume ≥ 500 ml	217 (71.1) *n* = 305	289 (65.1) *n* = 444	278 (49.5) *n* = 551	182 (28.1) *n* = 648	152 (28.4) *n* = 536	< 0.0001
Largest volume observed during hospital stay	606 (188) 600 (500; 700) *n* = 287	589 (190) 500 (450; 700) *n* = 419	536 (192) 500 (400; 600) *n* = 515	457.6 (159.7) 400 (350; 500) *n* = 602	459.2 (156.1) 400 (400; 500) *n* = 497	< 0.0001
Indwelling urinary catheter	114 (28.1)	229 (35.0)	396 (56.6)	523 (75.7)	493 (78.8)	< 0.0001
Straight in-out catheterisation	150 (36.9)	156 (23.8)	76 (10.9)	27 (3.9)	17 (2.7)	< 0.0001
Indwelling and straight in-out catheterisation	142 (35.0)	270 (41.2)	228 (32.6)	141 (20.4)	116 (18.5)	< 0.0001
**Indwelling and straight in-out catheterisation + indwelling catheter**	***n*** **= 256**	***n*** **= 499**	***n*** **= 624**	***n*** **= 664**	***n*** **= 609**	
IDC days	4.16 (3.95) 3 (2; 5)	4.39 (4.47) 3 (2; 5)	4.92 (5.16) 3 (2; 6)	3.90 (3.10) 3 (2; 5)	3.58 (3.26) 3 (2; 4)	0.11
IDC re-insertion	32 (12.5)	78 (15.6)	110 (17.6)	76 (11.4)	74 (12.2)	0.083
IDC present on discharge Missing	23 (9.0)	56 (11.2)	85 (13.6) 1	60 (9.0)	49 (8.1)	0.095
Straight in-out catheterisation^a^	3.11 (3.17) 2 (1; 4) *n* = 292	2.79 (2.58) 2 (1; 4) *n* = 426	2.07 (1.58) 1 (1; 3) *n* = 304	1.68 (1.04) 1 (1; 2) *n* = 168	1.92 (1.43) 1 (1; 2) *n* = 133	< 0.0001
**Documentation**	***n*** **= 256**	***n*** **= 499**	***n*** **= 624**	***n*** **= 664**	***n*** **= 609**	
Urine volume when inserting an in-dwelling catheter	17 (6.6)	4 (0.8)	335 (53.7)	535 (80.6)	452 (74.2)	< 0.0001
Documented indication	117 (45.7)	201 (40.3)	387 (62.0)	528 (79.5)	472 (77.5)	< 0.0001
Documented removal plan	17 (6.6)	38 (7.6)	147 (23.6)	378 (56.9)	225 (36.9)	< 0.0001

For categorical variables, n (%) is presented

For continuous variables, the mean (SD)/median (Q1; Q3)/*n* = is presented

For comparisons between groups, the Mantel-Haenszel chi-square test was used for ordered categorical variables

The Jonckheere-Terpstra test was used for continuous variables

^a^Both straight in-out and indwelling + straight in-out catheterisation treatment groups

The odds of contracting bladder distension were reduced by 40% per year and did not differ substantially after adjustment for age, gender, hospital LOS and ASA-classification score (Table 4). The univariable logistic regression analysis showed that the odds of bladder distension were higher in men and when having a longer hospital LOS. The multivariable regression analysis shows that the years of the intervention, gender and hospital LOS were independent risk factors (Table 4).

**Table 4:** Uni- and multivariable regression for the event of bladder distension

				Univariable^**a**^			Multivariable^**b**^	
**Variable**	**n missing**	**Value**	**n (%) of event**	**OR (95%CI) bladder distension**	***p*-value**	**Area under ROC curve (95%CI)**	**OR (95%CI) bladder distension**	***p*-value**
**Years**	0	**Year 1**	165 (40.6)					
		**Year 2**	211 (32.2)					
		**Year 3**	154 (22.0)					
		**Year 4**	84 (12.2)					
		**Year 5**	57 (9.1)	0.60 (0.56–0.64)	< 0.0001	0.68 (0.66–0.70)	0.61 (0.57–0.66)	< 0.0001
**Gender**	0	**Female**	447 (20.5)					
		**Male**	224 (24.9)	1.28 (1.07–1.54)	0.0082	0.53 (0.51–0.55)	1.28 (1.05–1.56)	0.013
**Age**^**+**^	0	**65–80**	208 (21.1)					
		**81–88**	233 (21.1)					
		**89–104**	230 (23.3)	1.06 (0.95–1.18)	0.27	0.52 (0.49–0.54)	1.08 (0.96–1.21)	0.21
**Hospital Length of stay**^**++**^	0	**3–8**	158 (16.5)					
		**9–13**	228 (19.8)					
		**14–68**	285 (29.5)	1.36 (1.24–1.48)	< 0.0001	0.59 (0.57–0.62)	1.16 (1.05–1.27)	0.0019
**Diabetes**	0	**yes**	110 (23.1)					
		**no**	561 (21.6)	0.91 (0.72–1.15)	0.45	0.51 (0.49–0.52)	0.89 (0.70–1.14)	0.36
**ASA score**	0	**1**	13 (15.3)					
		**2**	268 (21.8)					
		**3**	353 (22.4)					
		**4**	37 (19.6)	1.03 (0.90–1.18)	0.64	0.50 (0.48–0.53)	0.96 (0.83–1.10)	0.55

*P*-values, OR and area under ROC curve were based on original values and not on stratified groups

*OR * the ratio of the odds of an increase in the predictor of one unit

^+^OR is the ratio of the odd of an increase in the predictor of ten units and ^++^of seven units

^a^All tests were performed with univariable logistic regression

^b^Multivariable logistic regression model including: years, gender, age, hospital length of stay, diabetes mellitus and ASA-classification score. Area under ROC curve with 95% CI for multivariable model = 0.69 (0.67–0.71)

### Secondary outcomes

Changes in a urine volume ≥ 500 ml and largest observed urine volume is presented in Table 3. The mean difference over the years in urine volume ≥ 500 ml were 42.8 (95% CI 36.2;49.4) and largest volume 147.3 (95% CI, 122.7; 171.4). The odds of a urine volume of ≥500 ml were reduced by 42% yearly, OR 0.58, (95% CI 0.54–0.62, *p* < 0.0001) and did not differ substantially after adjustment for age, gender, hospital LOS and ASA-classification score (Additional file 3). The multivariable regression analysis shows that years 1–5, LOS and ASA-classification score were all independent risk factors to urine volume of ≥500 ml (Additional file 3).

Significant improvements were found over the 5 years, in documentation related to catheter indication, the present of a removal plan and urine volume when inserting the IDC (Table 3). We found more patients with a first IDC insertion earlier in the patient pathway (Additional file 4). Significant reductions were found in patients treated with both an IDC and straight in-out catheterisation during hospital stay, as well as the number of straight in-out catheterisations. We found no significant reductions in IDC days, re-catheterisation and catheter presented at discharge over the years.

### Findings related to the nurse-driven urinary catheterisation protocol

The RN assessed 586 patients for 10 months. Of these, 544 patients had documented IDC indication(s) of which most were correctly assessed. The most common indications were related to UR/residual urine, morbidity or to ensure haemodynamic stability (Additional file 5). Patient involvement and removal plan is presented in Additional file 6.

## Discussion

We found that the intervention was associated with a reduced incidence of bladder distension over 5 years, from 4 to 1 in 10 patients, and the mean yearly incidence of patients with a bladder volume of ≥500 ml was almost halved. The incidence of bladder distension was high the first 2 years. The findings from other studies using the same trigger in orthopaedic patients found bladder distension in a small percentage of patients [5, 16, 58]. However, comparisons with our study are problematic, due to the different study design and case mix. Further, the incidence of a urine volume of ≥500 ml is in the higher range in year 1, while those in years 4 and 5 are in the lower ranges, when compared with other studies [20, 22, 23]. Still, comparison with other studies is difficult as the definition of UR differ. Moreover, contrary to Adunsky et al. [22], we found that diabetes did not predict bladder distension or a urine volume of ≥500 ml in our cohort.

### The intervention

We have not found any theory-driven intervention similar to ours to reduce bladder distension or UR. Most nurse-driven protocols have been shown to reduce both the length and use of IDC, associated UTIs and catheter trauma, by using appropriate indications, removal plans and timely bladder scanning [33, 59–62]. We found that more patients received a catheter over the intervention years and in parallel a decrease he incidence of UC-UTI [27]. This despite no significant reductions in IDC days and re-catheterization rates. It is possible that implementing aseptic insertion techniques and antiseptic prewash to some extent counter the development of bacteriuria. A review by, Zhang et al.’s [63] supports the use of a short-term IDC, removed within 24–48 hours, in preventing post-operative UR compared with straight in-out catheterisation, without increasing the risk of UC-UTI. Moreover, our study confirms the importance of using timely measurement of residual urine, starting in the ED to reduce the risk of overfilling the bladder [13, 15, 30]. Considering the decreased number of straight in-out catheterisation and that the catheter was inserted earlier in the care pathway, the intervention might have contributed to timelier insertion of IDC and thereby avoiding unnecessary catheterisations. Further, the intervention significantly improved RNs catheter related documentation and confirmed the lack in documentation among both RNs and physicians [64, 65].

To facilitate for RNs to rethink and relearn “new” catheter best practices through “embracing” doubts as well as allowing participants to examine the problem from different perspectives [37, 38], seems to be a way forward in preventing bladder distension in hip fracture patients. Our findings support the belief that bladder distension is a nurse-sensitive adverse event [58] and, by allowing nurses to initiate catheter treatment, through pre-defined clinical decision tool, a timelier catheter insertion can be facilitated. Moreover, we agree with Rutberg et al. [5] that avoiding an IDC to prevent a UTI might increase the risk of bladder distension and that preventive strategies ought to address both types of adverse events. Further, either routine insertion of an IDC or the strict use of straight in-out catheterisation may be recommended in this patient group [17, 66, 67]. Instead, an individual assessment of each patient is important [44, 46, 49–52].

### Strength and limitations

The strength of our study is the large cohort size and the longitudinal observation period with continuous validation of the data. However, it is a single-centre study using outcome data from a specific patient cohort and we have not controlled for all potential confounding factors that could have affected our outcomes. For example, UR or residual urine on admission, or comorbidities such as Parkinson’s and stroke which increases the risk of lower urinary tract problems as these data were not available in the register. The lack in follow up after discharge is also a limitation. Moreover, LOS is difficult to interpret, as changes related to LOS may have several other explanations, such as changes in discharge routines or other adverse events. Further, urinary retention has been shown to increase hospital LOS in orthopaedic patients [68, 69] but not in hip fracture patients [17, 22]. We did not include UC-UTI as a covariate as it is difficult to single out the dependency between UTI and UR.

The initial lower completeness of data can be regarded as a limitation. During the first year, the register suffered from organisational issues and the completeness was approximately 50–60% if we anticipated a yearly incidence of 800–900 hip fracture patients. However, the yearly incidence also includes those admitted to the orthopaedic wards and thereby not reported to the register.

## Conclusion

This study provides new insights in how an intervention which includes the co-creation of a nurse-driven UC-protocol and timely bladder scanning schedule can reduce bladder distension and urine volume ≥ 500 ml in patients with hip fracture. The findings suggest that RNs, in line with their core competencies, can use supporting tools to ensure timely and appropriate catheter insertion. Still more studies are needed to investigate if this approach is applicable to other settings.

## Supplementary Information

**Additional file 1.** Example of patient case using SBAR pre-intervention.

**Additional file 2.** Brief nurse-driven urinary catheterisation protocol.

**Additional file 3.** Uni- and multivariable regression in the event of a urine volume of ≥500 ml.

**Additional file 4.** Setting for first insertion of indwelling urinary catheter.

**Additional file 5. **Identified and documented catheter indications, *N* = 586.

**Additional file 6.** Patient involvement, seeking support and removal plan.

## Data Availability

The summary data are available in the main document. We have no ethical approval to share the datasets generated and analysed during the current study. If anyone wishes to request the datasets from this study, the corresponding author can be contacted.

## Abbreviations

CI: Confidence interval
CRM: Crew Resource Management
ED: Emergency Department
ICU: Intensive care unit
IDC: Indwelling urinary catheter
LOS: Length of stay
OD: Operating room
OR: Odds ratio
PACU: Post-anaesthesia care units
SD: Standard Deviation
UC: Urinary catheter
UR: Urinary retention
