# Supplementary material for: A co-created nurse-driven catheterisation protocol can reduce bladder distension in acute hip fracture patients - results from a longitudinal observational study
Source: BMC Nurs. 2022 Oct 12;21:276. doi: 10.1186/s12912-022-01057-z (PMC9559039; doi:10.1186/s12912-022-01057-z)
Supplement: Supplementary file 1 — Additional file 1. Example of patient case using SBAR pre-intervention. [file 12912_2022_1057_MOESM1_ESM.docx]

| **Additional file 1. Example of patient case using SBAR pre-intervention** |
| --- |
| **S-Situation** Female, 83 years of age, fall at home, femur fracture, admitted to the emergency department (ED) and scheduled for surgery within 24 hours  **B-Background** Diabetes, hypertension, no homecare  **A-Assessment:** Stable vital parameters and normal laboratory test, urinary retention on admission  **R-Recommendation**: Primary measure: straight in-out catheterisation was performed in the ED, verbally reported to register nurses on the ortho-geriatric ward. No documentation of straight in-out catheterisation or urine volume in the RETTS (Rapid Emergency Triage and Treatment System) or the electronic medical record from the stay in the ED.  Secondary measures in further care: Three straight in-out catheterisations on the ward, 600-800 ml before surgery. Indwelling urinary catheter inserted in the operating room due to urinary retention. Catheter removed post-operative day 1, as routine. Thereafter five straight in-out catheterisations with a volume of between 500-700 ml, re-catheterised on day four. Six-day hospital stay. Physician discharge notes: catheter to be removed in the short-stay rehabilitation unit with a follow-up on bladder function, antimicrobial treatment for urinary tract infections.  Could we have done anything differently? |
