# Supplementary material for: A co-created nurse-driven catheterisation protocol can reduce bladder distension in acute hip fracture patients - results from a longitudinal observational study
Source: BMC Nurs. 2022 Oct 12;21:276. doi: 10.1186/s12912-022-01057-z (PMC9559039; doi:10.1186/s12912-022-01057-z)
Supplement: Supplementary file 2 — Additional file 2. Brief nurse-driven urinary catheterisation protocol. [file 12912_2022_1057_MOESM2_ESM.docx]

|  |  |  |  |  |  |  |  |  |  |  |
| --- | --- | --- | --- | --- | --- | --- | --- | --- | --- | --- |
| **Additional file 2. Brief nurse-driven urinary catheterisation protocol** | | | | | | | | | | |
| **ID_____**  **Perform a thorough patient using the hospital patient assessment tools. Use the timely bladder scanning schedule. Consider previous bladder issues and the further care.** |  | Ward/  date/  time | Catheter  Indication | Number of  Intermittent  Catheterisation | Seeking support from colleague and/or physician | Patient participation  Yes/No | Chronic catheter, intermittent  Self-catheterisation,  Urostomy/suprapubic  catheter | Removal  date | Catheter removed | Re-catheter,  Indication |
| 1.Acute urinary retention and/or blood clot/haematuria.  2. Haemodynamic instability, in need of intravenous fluid and urine output measurement.  3. Renal dysfunction – in need of urine output measurement.  4. In need of intravenous diuretics  5. Preoperative residual urine ≥ 200ml with risk of bladder distension in pre-operative bladder scan  6. An anticipated time of more than three hours from pre-operative void, to end of surgery.  7. Previous bladder damage or neurogenic bladder dysfunction  8. End-of-life care  9. Unable to void related to severe illness, e.g. multiple fractures  10. Incontinence with risk of contamination of perineal and sacral areas and wound areas |  |  |  |  |  |  |  |  |  |  |
|  |  |  |  |  |  |  |  |  |  |  |
|  |  |  |  |  |  |  |  |  |  |  |
|  |  |  |  |  |  |  |  |  |  |  |
|  |  |  |  |  |  |  |  |  |  |  |
|  |  |  |  |  |  |  |  |  |  |  |
|  |  |  |  |  |  |  |  |  |  |  |
| **Removal plan**  A. Within 24-48 hours, B. Physician’s order, e.g. suspected bladder damage or deteriorated patient, C. Daily evaluation of catheter requirement | | | | | | | | | | |
| **Commentary:** | | | | | | | | | | |
