# Supplementary material for: A co-created nurse-driven catheterisation protocol can reduce bladder distension in acute hip fracture patients - results from a longitudinal observational study
Source: BMC Nurs. 2022 Oct 12;21:276. doi: 10.1186/s12912-022-01057-z (PMC9559039; doi:10.1186/s12912-022-01057-z)
Supplement: Supplementary file 3 — Additional file 3. Uni- and multivariable regression in the event of a urine volume of ≥500 ml. [file 12912_2022_1057_MOESM3_ESM.docx]

| **Additional file 3. Uni- and multivariable regression in the event of a urine volume of ≥ 500ml** | | | | | | | | |
| --- | --- | --- | --- | --- | --- | --- | --- | --- |
|  | | | | **Univariable*** | | | **Multivariable**** | |
| **Variable** | **n missing** | **Value** | **n (%) of event** | **OR (95%CI) ≥ 500 ml** | ***p*-value** | **Area under ROC curve (95%CI)** | **OR (95%CI) ≥ 500 ml** | ***p*-value** |
| **Year** | 0 | **Year 1** | 217 (71.1) |  |  |  |  |  |
|  |  | **Year 2** | 289 (65.1) |  |  |  |  |  |
|  |  | **Year 3** | 278 (50.5) |  |  |  |  |  |
|  |  | **Year 4** | 182 (28.1) |  |  |  |  |  |
|  |  | **Year 5** | 152 (28.4) | 0.58 (0.54-0.62) | <0.0001 | 0.69 (0.67-0.71) | 0.59 (0.55-0.64) | <0.0001 |
| **Gender** | 0 | **Female** | 778 (44.4) |  |  |  |  |  |
|  |  | **Male** | 340 (46.6) | 1.09 (0.92-1.30) | 0.31 | 0.51 (0.49-0.53) | 1.10 (0.91-1.33) | 0.31 |
| **Age^+^** | 0 | **65-80** | 353 (45.0) |  |  |  |  |  |
|  |  | **81-88** | 387 (44.0) |  |  |  |  |  |
|  |  | **89-104** | 378 (46.0) | 1.01 (0.91-1.11) | 0.86 | 0.50 (0.48-0.53) | 1.01 (0.90-1.12) | 0.90 |
| **Hospital length of stay^++^** | 0 | **3-8** | 305 (38.8) |  |  |  |  |  |
|  |  | **9-13** | 396 (42.4) |  |  |  |  |  |
|  |  | **14-68** | 417 (54.7) | 1.36 (1.24-1.49) | <0.0001 | 0.58 (0.56-0.60) | 1.14 (1.03-1.25) | 0.010 |
| **Diabetes mellitus** | 0 | **yes** | 162 (43.0) |  |  |  |  |  |
|  |  | **no** | 956 (45.4) | 1.10 (0.88-1.37) | 0.39 | 0.51 (0.49-0.52) | 1.05 (0.83-1.33) | 0.70 |
| **ASA** | 0 | **1** | 27 (39.1) |  |  |  |  |  |
|  |  | **2** | 477 (47.6) |  |  |  |  |  |
|  |  | **3** | 559 (44.1) |  |  |  |  |  |
|  |  | **4** | 55 (37.9) | 0.89 (0.79-1.01) | 0.077 | 0.52 (0.50-0.54) | 0.85 (0.74-0.98) | 0.022 |
| P-values, OR and area under the ROC curve are based on original values and not on stratified groups. OR is the ratio of the odds of an increase in the predictor of one unit.  ^+^ OR is the ratio of the odd of an increase in the predictor of ten units and ^++^of seven units.  *) All the tests are performed with univariable logistic regression. **) Multivariable logistic regression model including year, gender, age, hospital length of stay, diabetes mellitus and ASA-classification score. Area under the ROC curve with 95% CI for multivariable model = 0.70 (0.67-0.72) | | | | | | | | |
