# Supplementary material for: A co-created nurse-driven catheterisation protocol can reduce bladder distension in acute hip fracture patients - results from a longitudinal observational study
Source: BMC Nurs. 2022 Oct 12;21:276. doi: 10.1186/s12912-022-01057-z (PMC9559039; doi:10.1186/s12912-022-01057-z)
Supplement: Supplementary file 4 — Additional file 4. Setting for first insertion of indwelling urinary catheter. [file 12912_2022_1057_MOESM4_ESM.docx]

| Additional file 4. Setting for first insertion of indwelling urinary catheter | | |
| --- | --- | --- |
| Setting | Year 1 | Year 5 |
| Emergency department | 45/256, (17.6) | 178/609 (29.2) |
| Ortho-geriatric wards | 76/256, (29.7) | 299/609, (49.1) |
| Operating room department | 116/256, (45.3) | 127/09, (20.8) |
| Postoperative care unit/Intensive care unit | 19/256, (7.4) | 4/609, (0.3) |
| Number and (percent) | | |
