# Supplementary material for: A co-created nurse-driven catheterisation protocol can reduce bladder distension in acute hip fracture patients - results from a longitudinal observational study
Source: BMC Nurs. 2022 Oct 12;21:276. doi: 10.1186/s12912-022-01057-z (PMC9559039; doi:10.1186/s12912-022-01057-z)
Supplement: Supplementary file 5 — Additional file 5. Identified and documented catheter indications, N = 586. [file 12912_2022_1057_MOESM5_ESM.docx]

| **Additional file 5. Identified and documented catheter indications, N=586** | | |
| --- | --- | --- |
| **Indication, number (%)** | **N=586^+^** | **More than one indication^++^**  **n=184** |
| Acute urinary retention and/or blood clot/haematuria | 75 (12.8) | 46 (25.0) |
| Haemodynamic instability, in need of intravenous fluid and urine output measurement | 66 (11.3) | 45 (24.4) |
| Renal dysfunction – in need of urine output measurement | 9 (1.5) | 30 (16.3) |
| In need of intravenous diuretics | 2 (0.3) | 12 (6.5) |
| Preoperative residual urine ≥ 200ml with risk of bladder distension in pre-operative bladder scan | 127 (21.7) | - |
| An anticipated time of more than three hours from pre-operative void, to end of surgery | 13 (2.2) | - |
| Previous bladder damage or neurogenic bladder dysfunction | 1 (0.2) | - |
| End-of-life care | - | 3 (1.6) |
| Unable to void related to severe illness e.g multiple fractures | 50 (8.5) | 43 (23.3) |
| Incontinence with risk of contamination of perineal and sacral areas and wound areas | 2 (0.3) | 5 (2.7) |
| More than one indication ^++^ | 184 (31.4) |  |
| Anaesthesiologists ordered IDC (not a predefined indication) | 7 (1.2) |  |
| Incorrect indication* | 2 (0.3) |  |
| Catheter inserted without documentation in the protocol or medical record* | 6 (1.0) |  |
|  | | |
| Intermittent catheterisation  Refused IDC, despite indication. Straight in-out catheterisation with continuous voiding problem at discharge | 19 (3.2)  1 (0.2) |  |
| -No indication for IDC or straight in-out catheterisation  -Unable to insert catheter despite indication (frequent bladder scanning, managed without catheterization) | 21 (3.6)  1 (0.2) |  |
| Abbreviations: IDC= Indwelling urinary catheter, * All had indications. | | |
