# Supplementary material for: A co-created nurse-driven catheterisation protocol can reduce bladder distension in acute hip fracture patients - results from a longitudinal observational study
Source: BMC Nurs. 2022 Oct 12;21:276. doi: 10.1186/s12912-022-01057-z (PMC9559039; doi:10.1186/s12912-022-01057-z)
Supplement: Supplementary file 6 — Additional file 6. Patient involvement, seeking support and removal plan. [file 12912_2022_1057_MOESM6_ESM.docx]

| **Additional file 6. Patient involvement, seeking support and removal plan** | |
| --- | --- |
| **Patient involvement, number (%)** | **N=586** |
| Yes | 286 (48.9) |
| No | 15 (2.5) |
| Not reported | 245 (41.8) |
| Cannot participate related to cognitive dysfunction | 17 (2.9) |
| Language difficulties | 2 (0.3) |
| Not applicable | 21 (3.5) |
| **Seeking Support*** | |
| RN | 15 (2.6) |
| Physician | 43 (7.4) |
| RN or Physician | 222 (37.8) |
| Not reported | 226 (38.6) |
| No need to discuss | 80 (13.6) |
| **Removal plan** | **n=544** |
| 24-48 hours | 324 (59.5) |
| Indication no longer presents | 11 (1.9) |
| Physician order | 40 (7.4) |
| No removal plan | 146 (26.8) |
| After surgery (not a predefined removal plan) | 21 (3.8) |
| Uncertain (not a predefined removal plan) | 2 (0.4) |
| **Removed according to plan** | |
| Yes | 196 (36.0) |
| No | 341 (62.7) |
| Self-terminated | 7 (1.3) |
| **Reasons for re-catheterisations** | **n=66** |
| Urinary retention or residual urine  Hip-dislocation (patient had urinary retention) | 4 (72.7)  1 (0.1) |
| Deterioration | 8 (12.1) |
| Self-terminated | 3 (4.5) |
| Need of bigger size catheter | 2 (3.0) |
| Re-operation | 2 (3.0) |
| **Physician documentation n=544** | |
| Indication | 61 (11.2) |
| Removal plan | 0 |
| Abbreviation: RN=Registered nurse,  *Nurse discussed catheter treatment with a peer or physician. | |
